# Atypical presentation of oculogyric crisis induced by atypical antipsychotics: Causes and management

**DOI:** 10.1192/j.eurpsy.2026.11787

**Published:** 2026-07-31

**Authors:** Y. Ben Othmene, A. Aissa, S. Aouadi, A. Hlaoui, R. Hosni, A. Ouertani, R. Jomli

**Affiliations:** Avicenne psychiatry department, Razi Hospital, Tunis, Tunisia

## Abstract

**Introduction:**

Oculogyric crisis (OGC) is a rare focal dystonia of the ocular muscles, characterized by involuntary upward eye movements and often accompanied by anxiety. OGC are rarely reported with atypical antipsychotics and generally respond to anticholinergic agents.

**Objectives:**

The aim is to illustrate an atypical presentation of Olanzapine induced OGC and its management.

**Methods:**

This case report was compiled through clinical observation and a review of the patient’s medical history. Symptom progression was monitored through psychiatric evaluations with medication adjustments made based on severity and tolerability.

Informed consent was obtained and confidentiality was maintained.

**Results:**

We report the case of a 22-year old female with no personal medical history, but a family history of intellectual deficiency and bipolar disorder. The patient was diagnosed with schizophrenia at the age of 18 and initially treated with Risperidone at a dose of 3 mg/d, resulting in significant improvement. However, after discontinuing treatment, she had a relapse, manifesting as persecutory delusions, Kandinsky–Clérambault syndrome with intrapsychic hallucinations, thought broadcasting and a sensation of being controlled by external forces. She sought psychiatric care and was restarted on Risperidone at a dose of 4 mg/d. Shortly after, she developed akathisia, which was attributed to Risperidone treatment after a neurological consultation ruled out an organic cause.

Therefore, Risperidone was discontinued, and the patient was switched to Olanzapine 15 mg/d. Four months later, the patient developed OGC with intermittent up-rolling of eyeballs, anxiety and third-person intrapsychic hallucinations of derogatory content concomitant with the episodes. This was partially managed with Bipiriden at 8mg/d. However, the crises persisted, leading to the introduction of Propranolol at 20mg/d, which resulted in a notable improvement in the OGC. The frequency of distressing episodes decreased to three to four brief occurrences per month. The psychotic symptoms also came under control.

**Conclusions:**

This case highlights an atypical presentation of OGC in a patient with schizophrenia, induced by an atypical antipsychotic. It emphasizes the importance of recognizing OGC in psychiatric patients and the need for careful treatment adjustments to improve treatment compliance and quality of life.

**Disclosure of Interest:**

None Declared

